# The abundance of the potential pathogen *Staphylococcus hominis* in the air microbiome in a dental clinic and its susceptibility to far‐UVC light

**DOI:** 10.1002/mbo3.1348

**Published:** 2023-03-05

**Authors:** Marilena Aquino de Muro, Igor Shuryak, Anne‐Catrin Uhlemann, Alice Tillman, Dwayne Seeram, Joseph Zakaria, David Welch, Steven M. Erde, David J. Brenner

**Affiliations:** ^1^ Center for Radiological Research, Columbia University Irving Medical Center New York New York USA; ^2^ Columbia University Irving Medical Center Microbiome Core Facility New York New York USA; ^3^ Columbia University College of Dental Medicine New York New York USA

**Keywords:** 222 nm‐far‐UVC susceptibility, air microbiome, air sampling, pathogenic, regression modeling, *Staphylococcus hominis*

## Abstract

The dental clinic air microbiome incorporates microbes from the oral cavity and upper respiratory tract (URT). This study aimed to establish a reliable methodology for air sampling in a dental clinic setting and quantify the abundance of culturable mesophilic aerobic bacteria present in these samples using regression modeling. *Staphylococcus hominis*, a potentially pathogenic bacterium typically found in the human oropharynx and URT, was consistently isolated. *S. hominis* was the most abundant species of aerobic bacteria (22%–24%) and comprised 60%–80% of all *Staphylococcus* spp. The study also assessed the susceptibility of *S. hominis* to 222 nm‐far‐UVC light in laboratory experiments, which showed an exponential surface inactivation constant of *k* = 0.475 cm^2^/mJ. This constant is a critical parameter for future on‐site use of far‐UVC light as a technique for reducing pathogenic bacterial load in dental clinics.

## Graphical abstract

This study established a reliable methodology for air sampling in a dental clinic setting and quantified the abundance of culturable mesophilic aerobic bacteria present in these samples using regression modeling. *Staphylococcus hominis*, a potentially pathogenic bacterium, was the most abundant species of aerobic bacteria and comprised 60%–80% of all *Staphylococcus* spp. in the clinic air microbiome. The study also assessed the susceptibility of *S. hominis* to 222 nm far‐UVC light suggesting far‐UVC light for reducing bacterial load in dental clinics.

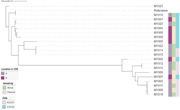

## 1 INTRODUCTION

The air microbiome incorporates aerobic and anaerobic microbes from the oral cavity and upper respiratory tract (URT). They are expelled by breathing, talking, singing, coughing, and sneezing, as well as during periods of dental treatment and procedures. Procedures in a dental clinic such as tooth preparation, tooth drilling, ultrasonic scaling, tooth polishing, and tooth extraction can all result in the production of aerosols and saliva splatter, each of which propels oral bacteria, commensal and/or pathogenic, into the air (Adhikari et al., 2017; Decraene et al., 2008). Unclean air conditioning and heating systems, as well as the walk‐in public, can also contribute to the microbial load as they carry bacteria and fungi present in the dust outside (Alabdalall et al., 2019). Multichair dental clinics, where patients are treated next to each other within the same room and indoor air, can promote the cross‐transmission of potentially pathogenic bacteria to other patients who are undertaking dental treatment in this setting (Kimmerle et al., 2012).

The indoor air microbiome of dental clinics is understudied and data on the potentially high loads of oral pathogens being significant contributors is not readily available in the literature. It has been reported that periodontal infections are mostly caused by nonsporulating obligate anaerobic bacteria, such as *Fusobacterium nucleatum* (Gram‐negative) *Prevotella intermedia* (Gram‐negative), *Actinomyces israelii* (Gram‐positive) and facultative anaerobic Gram‐positive cocci such as *Streptococcus sanguinis* and other oropharyngeal *Streptococcus* Anginosus Group (SAG) being the most common isolates. Although these bacterial species are considered to be a normal part of the microbiota of the oral cavity and gastrointestinal tract, they have also been reported to cause infections and systemic diseases (Han et al., 2000; Jiang et al., 2020; Martini et al., 2020). For example, SAG bacteria have been isolated from the sputa of patients with cystic fibrosis, chronic obstructive pulmonary disease, and bronchiectasis (Waite et al., 2012). The significance of this study is to find out if there are pathogenic bacteria, such as SAG bacteria, in the dental clinic air microbiome which could be transmitted by air and therefore be a contributing factor to respiratory infections in immunocompromised or elderly patients who are undergoing dental care at the clinic.

*Staphylococcus epidermidis* is one of the most abundant bacteria in the nasal cavity and can prevent the colonization of the nostrils with respiratory pathogens (Ortega‐Peña et al., 2022). It is also reported in the literature that *Staphylococcus hominis* is the second most frequently isolated Coagulase‐Negative *Staphylococcus* species (CoNS) from healthy skin, and there is emerging evidence to suggest that it may play a significant role in excluding pathogens, including *Staphylococcus aureus*, from colonizing or infecting the skin (Severn et al., 2022).

Conversely, *S. hominis* has been reported as an opportunistic pathogenic bacterium typically found in the human oropharynx and URT (Otto, 2010; Szczuka et al., 2018); present in the blood of immunocompromised patients with bacteremia, septicemia, endophthalmitis, and endocarditis (Ahmed et al., 2017; Jeon et al., 2022; Muraki et al., 2022; Uddin et al., 2022; Vasconcellos et al., 2022), and often it can form biofilms on medical devices (Villarreal‐Salazar et al., 2022).

This study aimed to establish a protocol for sampling indoor air in a dental clinic and assessing the diversity of the air microbiome. This study aimed to use its findings to predict the makeup of the air microbial environment in the clinic using regression modeling and statistical analysis. The focus was on a baseline for the most abundant culturable aerobic microbial species with relevance to the oral microbiology of the oropharynx and URT, which are potentially transmitted through the air. The susceptibility of the most abundant of the isolated bacterial species to 222 nm‐far‐UVC light was also assessed in laboratory experiments, to use far‐UVC light to reduce the number of those potentially pathogenic bacteria found in the dental clinic in future studies.

## 2 MATERIALS AND METHODS

### 2.1 Active samplings and microbial culturing

Air samples were collected at two distinct locations within the Vanderbilt Dental Clinic at the Columbia University College of Dental Medicine, New York City, NY. This dental clinic used has approximately 4300 ft^2^, and a 10 ft high ceiling. The total number of people in the room was counted every 30 min, that is, twice an hour throughout the total period in which the air samplings were taken in the dental clinic. Although the dental clinic has two entrances, patients gain access to the treatment room using only one entrance. The maximum number of chairs for dental treatments is 24. The dentists always wore two masks, a KN95 plus a surgical mask, according to standard protocol in the dental clinic. Patients had 1‐, 2‐, or 3‐h appointment slots, and were not wearing masks while undergoing dental treatment. The dental clinic has a variable air volume (VAV) heating, ventilation, and air conditioning (HVAC) system for ventilation and temperature control within the space. All of the supply air diffusers and return grilles for the VAV HVAC system are located within the ceiling. The HVAC system utilizes a single 12,000 cfm air handling unit for the clinical area where sampling occurred. The volume of air supplied for the clinic equates to approximately 10 air changes per hour (ACH). The supply is filtered using air filters with a minimum efficiency reporting a value of 14 (MERV14).

The two locations within the dental treatment area used for air samplings were over two 1.5 m in height cabinets situated in the center of the dental clinic open‐space room. These locations were chosen to assess patients' microbial load contribution while they receive dental care. Samples were taken during a period of either 3 or 4 h while the clinic was open to patients and on 2 days of each week between November 2021 and March 2022. Concomitantly active air sampling and passive air sampling were used, as follows.

For the active air sampling, one Sartorius MD8 Airport was used, which was fitted with a 9 cm gelatin filter and operated at an airflow rate of 50 L/min. Air samplings were performed following this protocol in November 2021: the first filter for 15 min, a second filter for 1 h, then a third filter for 2 h; in December 2021: the first filter for 1 h, then a second filter for 3 h. Following analysis of initial results, the regime was changed to one filter for 1 h then a second filter for 3 h in one location. This later regime was followed in February and March 2022 active air samplings: the first filter for 1 h and a second filter for 3 h on each location. Table 1 summarizes all protocols used in this study.

**Table 1:** Protocols for air samplings used in this study.

Timeframe for air samplings (without UVC exposure) at the Vanderbilt Dental Clinic: Initially for initially 3 h, then 4 h from 9:30 AM through 1:30 PM		
November 2021	December 2021	February–March 2022
Tuesday + Thursday	Tuesday + Thursday	Thursday
*Active sampling*	*Active sampling*	*Active sampling*
(1 location in VC5)	(1 location in VC5)	(2 locations in VC5)
1st filter = 15 min	1st filter = 1st hour	1st filter = 1st hour
2nd filter = 1st hour	2nd filter = 2nd + 3rd + 4th hour	2nd filter = 2nd + 3rd + 4th hour
3rd filter = 2nd+ 3rd hour		
*Passive sampling*	*Passive sampling*	*Passive sampling*
(1 location in VC5)	(1 location in VC5)	(2 locations in VC5)
1‐h sampling × 3 h	1‐h sampling × 4 h	1‐h sampling × 4 h
10 plates/h × 1 location	10 plates/h × 1 location	20 plates/h × 2 locations

After active sampling, the gelatin filter was placed into a 50 mL conical centrifuge tube containing 10 mL of melted sterile Trypticase Soy Agar (TSA) 0.75%, this is the soft TSA top layer in this protocol.

TSA as a rich media would promote the growth of any viable but stressed bacteria cells propelled into the air as aerosols from dental procedures, physically injured due to the airport suction or floating for too long in the dental clinic air. No antibiotics were used for plating out active sampling filters because this study intended to assess the presence of fungal spores in the air.

**Figure 1:**
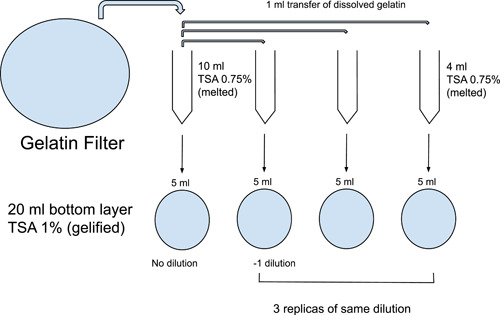
The two‐layer protocol developed and established in this study to plate out the 9‐cm gelatin filters fitted in the Sartorius MD8 airports (Germany), which were used to perform the active samplings in the Vanderbilt Dental Clinic at Columbia University.

This soft TSA had been kept in a water bath at 50°C to remain liquid. This 10 mL original stock (zero dilution) from each filter was kept in the water bath for 20 s to ensure all gelatin was dissolved. Then, 5 mL of this stock volume was poured onto a TSA 1% agar plate (bottom layer). The remaining 5 mL were aliquoted in 1 mL portions and transferred individually to 4 mL of liquid TSA 0.75% and then poured onto TSA 1% agar plates. Those 1‐mL aliquots are the 10^−1^ dilution from the original stock. All plates were left to set, inverted then incubated at 37°C for observation and counting of colony‐forming units (CFUs) in 72 h (Figure 1).

### 2.2 Passive samplings and microbial culturing

For the passive air sampling, 9‐cm media plates with 25 mL of TSA with 50 mg/L cycloheximide were used in one or two locations in the dental clinics: 10 plates in November/December 2021 in 1 location, or 20 media plates in February/March 2022 in 2 locations. Therefore, in those last 2 months, the total number of passive media plates used in the 4 h in two locations in the dental clinic was 160 plates per day of sampling. The two locations were on the top of two cabinets of just over 1.5 m in height in the center of the dental clinic. This set‐up follows the 1/1/1 scheme for passive sampling onto media plates (Pasquarella et al., 2000), that is, sampling for 1 h, at least 1 m from the floor, at least 1 m away from walls, or any obstacle. All passive sampling was performed with media plates left open for 1 h on top of cabinets “A” and “B.” The 20 plates per hour were arranged in a 4 × 5 grid which covered an area of 1522 cm^2^ on the top of each cabinet, equivalent to 1271 cm^2^ of the media surface. All passive media plates were incubated under aerobic mesophilic conditions at 37°C for 72 h. The use of TSA with antibiotics on passive plates was to avoid masking of bacterial count due to the overgrowth of fungal spores or possibly fungal inhibition of bacteria.

After incubation for both active and passive samplings, CFUs were counted, and representatives of various bacterial colony morphotypes were chosen, purified, and Gram‐stained.

### 2.3 Regression modeling and statistical analysis

The data collected from air sampling were compiled and imported into *R* 4.2.0 software for regression analysis (R Core Team, 2017). The dependent variable (outcome) was bacteria_CFUs (colony counts per sample). Since this is largely a proof‐of‐concept study, the combined colony counts for all bacteria were used to obtain larger numbers for statistical analysis to determine the major trends in the data.

The independent (predictor) variables were sampling (active or passive), month (calendar month of samplings), weekday (Tuesday or Thursday), hour (hour of samplings, 1, 2, or 3), location (on top of cabinet A or B) and people (average number of people in the room). The goal of the analysis was to construct a model for predicting the outcome variable (bacterial abundance) using the predictor variables. Bacterial CFU counts were significantly associated with two predictor variables: the day of the week (Tuesday or Thursday) and the number of people present in the dental clinic during sampling. At the dental clinic, Thursdays have patients' appointments from 8 AM to 3 PM, but Tuesdays only hold patients' appointments in the morning from 8 AM to 11 AM. Tuesday afternoons are reserved only for students to learn how to make dental prostheses, and they use human‐sized dummies for that. Details of the modeling procedure are described in Appendix.

In addition to the analysis of dental clinic air samples, this study also analyzed the survival data for the most abundant and relevant *Staphylococcus* spp. strains exposed to 222 nm‐far‐UVC light in laboratory experiments. An exponential dose–response model was used as described in the following equation, where *S* is the surviving fraction of bacterial CFUs, *D* is UV dose and *k* is the inactivation constant:

(1) ln[S]=−kD.

This study used linear regression to estimate parameter *k*, fitted by a robust procedure using the *rlm* function in the MASS *R* package. The slope of the regression line (*k* value) represents the sensitivity of each strain to inactivation by UVC, assuming an exponential dose response.

### 2.4 Bacterial identification using 16S **ribosomal RNA** (rRNA) gene

After colonies were chosen from active and passive sampling media plates, they were streaked onto fresh TSA plates for purification and isolation. Colonies were picked and resuspended in 30μL water. The 27F and 1492R primers were used to amplify the 16S rRNA gene using KAPA HiFi DNA Polymerase (Frank et al., 2008; Lane, 1991). Bands were visualized on a 1% agarose gel, and polymerase chain reaction products underwent Sanger Sequencing (Genomics from Azenta Life Sciences). Sequences were analyzed using Geneious Prime 2.0 and National Center for Biotechnology Information (NCBI) BLAST: Basic Local Alignment Search Tool (nih.gov)

### 2.5 *S. hominis* multilocus sequence typing (MLST) and whole genome sequencing (WGS) analyses

*S. hominis* isolated from active and passive samplings from cabinets A and B underwent WGS analysis. DNA was extracted using Illumina Flex Colony Lysis. Libraries were prepared using Nextera DNA Flex Library Prep kit (Illumina) and sequenced on a Miseq instrument (Illumina). MLST analysis was performed using SRST2 (v. 0.2.0) (Inouye et al., 2014).

To generate a full‐length genome for comparative analyses, isolate M1018, which was one of *S. hominis* isolated at the end of this 6‐month study, underwent long‐read sequencing with the Oxford Nanopore MinION platform (Oxford Nanopore). Base calling and demultiplexing of barcoded libraries were performed using MinKNOW (version 1.7.10) (Oxford Nanopore). The resulting reads were processed with Porechop (v 0.2.4) to remove adapters (Wick et al., 2017a) and were filtered with mothur for a minimum length of 5000 bp (Schloss et al., 2009). Hybrid assembly was performed using processed Nanopore long reads and Illumina short reads with unicycler (v0.5.0) tool (Wick et al., 2017b). Prokka (version 1.12) was used for open reading frame prediction and annotation of the resultant assembly (Seemann, 2014). A bandage was used to visualize de novo assembly and differentiate any chromosome versus plasmid contigs (Wick et al., 2015). Identification of mobile genetic elements was done with PHASTER and Island Viewer (Arndt et al., 2016; Bertelli et al., 2017).

After excluding mobile genetic elements and integrated phage regions, Illumina reads were mapped against the M1018 reference genome, and variant‐calling was performed using Snippy (v. 4.6.0) (Seemann, 2017). A Randomized Accelerated Maximum Likelihood (RAxML) (v 8.2.11) phylogenetic tree was generated using concatenated core chromosome SNPs in Geneious (v 10.0.09) (Kearse et al., 2012; Stamatakis, 2014). For RAxML tree generation, the nucleotide model was set to GTR GAMMA, and the algorithm was set to Rapid Bootstrapping and search for the best‐scoring maximum likelihood (ML) tree, with the number of bootstrap replicates set to 100 (Stamatakis, 2014). Isolate M1023 *S. hominis* subsp. *hominis* Type Strain ATCC 27844 was used to root the RAxML tree.

### 2.6 *Staphylococcus* spp. susceptibility to 222 nm‐KrCl‐far‐UVC excimer lamp

A 222 nm‐KrCl excimer lamp, emitting primarily at 222 nm, was used for laboratory testing for susceptibility. The lamp was operated with an optical bandpass filter to reduce the nonpeak emissions (Buonanno et al., 2021) and was used as a light source in laboratory experiments to assess the effectiveness of far‐UVC light to inactivate two of the most relevant *Staphylococcus* species identified as *S. hominis* and *S. epidermidis*. The three *S. hominis* isolates used for testing susceptibility to 222 nm‐far‐UVC were M1018, M1020, and M1022; and the two *S. epidermidis* isolates were A#2 and A#4; all of them isolated on February 24, 2022. Modified from Burlage (1998), chosen isolates were grown overnight in 50 mL Trypticase Soy Broth (TSB) in a shaker at 37°C/140 rpm. Then, 400 μL from this overnight culture were inoculated into 50 mL of fresh TSB, and cells were regrown to mid‐exponential phase for approximately 2.5–3 h until the absorption at 600 nm was verified to be between 0.3 and 0.4 using an Eppendorf Biophotometer D30. The cells were pelleted by centrifugation at 2103*g* for 8 min then resuspended to the same volume in phosphate‐buffered saline (PBS). Cells were centrifugated to a pellet again and resuspended again in 50 mL of PBS. A volume of 2 mL of this cell suspension was spread in a 4 cm diameter Petri dish swirling gently to cover the bottom of the plate. Dishes were exposed using the filtered excimer lamp at a distance of 20 cm. The irradiance at this distance was measured at 120 µW/cm^2^ using a Hamamatsu C9536 UV power meter with an H9535‐222 sensor head (Hamamatsu Corporation). Radiant exposure doses of 2, 10, or 20 mJ/cm^2^ were administered using respective exposure times of 17 s, 1 min 23 s, or 2 min 47 s. Plates were prepared in triplicate for each irradiation, and control plates remained unirradiated. Serial dilutions were prepared and 100 μL were spread onto TSA plates, incubated at 37°C in the dark, and colony forming units (CFUs) were counted after 48 h.

## 3 RESULTS AND DISCUSSION

### 3.1 Air sampling protocols, regression modeling, and statistical analysis

The diversity of culturable mesophilic bacteria on diverse air samples in the dental clinic was detected as shown in Table 3. When analyzing the training data using both NB and Poisson regression, the NB regression approach performed better by 126.4 AIC units. This is a significant difference in model support since each AIC unit represents an exp [1/2] fold change in relative likelihoods. The NB model was better supported because the distribution of bacterial counts in air samples was “over‐dispersed,” with a variance greater than the mean. This variability is likely due to variations in patient diagnoses, dental procedures, and bacterial burdens.

**Figure 2:**
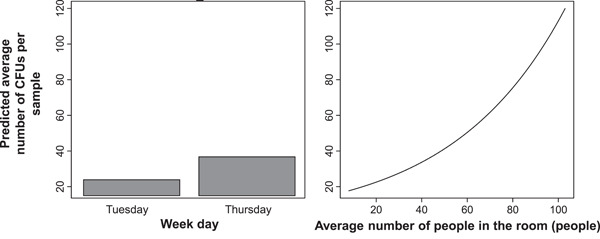
Behaviors of the best‐supported NB regression model for bacterial colony‐forming unit counts in dental clinic air samples. *y*‐axis = predicted bacterial colony count (returned to the linear scale from the natural log scale for better visualization). Each panel represents the effects of a specific predictor variable.

The NB regression model structure was identified by the model selection procedure. It retained only two predictors: weekday and people, in addition to the intercept. The model parameters were intercept = 2.833 ± 0.238 (standard error), *p* < 2 × 10^−16^; weekday (Tuesday vs. Thursday) = −0.439 ± 0.216, *p* = 0.042; people = 0.020 ± 0.005, *p* = 1.6 × 10^−4^ (Figure 2). The results suggest that the mean ln‐transformed bacterial colony count per sample was lower on Tuesdays than on Thursdays and was positively dependent on the number of people in the room. Thus, the study was able to show a correlation between the relevant microbial load input and the number of dental patients under treatment.

**Figure 3:**
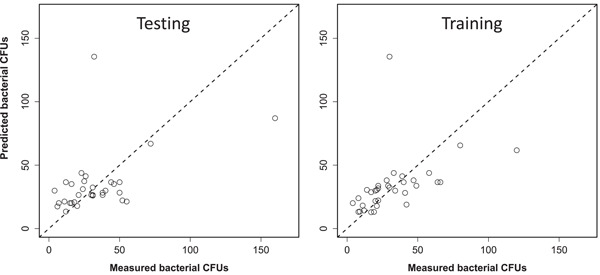
Comparisons of measured and predicted bacterial colony‐forming unit counts in dental clinic air samples. Predictions were made by the best‐supported NB regression model. Left: Results using the “testing” data; Right: Results using the “training” data.

NB regression outperformed Poisson regression for modeling bacterial abundance data based on AIC. The model performed well in predicting bacterial counts, considering the inherent data variability (e.g., differences in bacterial load in different patients). Its performance on a randomly selected training half of the data (using the ln scale) was: *R* ^2^ = 0.414, root mean square error (RMSE) = 0.567, mean absolute error (MAE) = 0.433. On the corresponding randomly selected testing half of the data, model performance was weaker, as expected: *R* ^2^ = 0.246, RMSE = 0.677, MAE = 0.509. A comparison of these numbers shows that the performance metrics were similar in training and testing data, suggesting that the model did not strongly overfit the training data set (Figure 3).

Model performance was evaluated in more detail using multiple training/testing splits of the data set. The random split was performed 300 times, fitted the model into each training split, and evaluated on each testing split. The results are summarized in Table 2. These results support the conclusion that performance using training and testing data did not differ dramatically. Such an outcome is expected because the model is quite simple (has only two predictors), so, it is unlikely to overfit the data. A potential interaction between these predictor variables was not statistically significant (*p* = 0.89).

**Table 2:** Best‐supported NB regression model's performance was evaluated using 300 random training/testing splits of the data set.

Performance metric	Training data		Testing data	
	Mean	SD	Mean	SD
*R* ^2^	0.345	0.098	0.317	0.104
RMSE	0.607	0.065	0.664	0.089
MAE	0.461	0.048	0.498	0.056

In addition to bacteria, the presence of fungi in air samples was also assessed. The fungal CFU counts, which were present on the two‐layer media plates without antibiotics for active sampling, were identified mostly as *Penicillium* spp. and *Aspergillus* spp. based on their typical colonial morphologies (Samsom et al., 1988). There was high variability in the fungal CFU counts, which is possibly attributed to the variable nature of the VAV HVAC system, which could vary the amount of airflow between assessment periods or days, assuming the fungal spores were loaded into the clinic air from the HVAC system.

The initial NB regression model with all tested predictors (weekday, hour, people, date, and month) had nonsignificant *p*‐values (0.20–0.79) for all these variables except month. The month was marginally significant at *p* = 0.041. Five other model variants were made by sequentially dropping the nonsignificant variables until only the intercept term was left. AIC scores for all variants were compared. That intercept‐only model was better by AIC scores than any of the other variants (by 0.3–5.1 units). This supports the conclusion that fungal abundance in clinic air samples is not driven by the presence of patients and dental procedures.

### 3.2 Air sampling protocols and bacterial identification

The active sampling allowed the isolation of CFUs per volume of indoor air: 20 bacterial CFUs per filter per 3000 L and 32 bacterial CFUs per filter per 9000 L of air on 14 March 2022, from the top of cabinet A; on the same date and time of samplings, from cabinet B, four bacterial CFUs per filter per 3000 L and 30 bacterial CFUs per filter per 9000 L of air.

Meanwhile, the passive sampling provided a snapshot of every hour throughout the sampling period, for example, 58 bacterial CFUs per 20 plates per 1271 cm^2^ of media surface on top of cabinet A on the last hour of air sampling on the same date; and 23 bacterial CFUs per 20 plates per 1271 cm^2^ of media surface on the top of cabinet B on the same date and time of passive samplings.

TSA also promoted pigment formation with the environmental bacteria *Microbacterium paraoxydans* and the skin bacteria *Kocuria* spp. exhibiting various shades of yellow contrasting to *Staphylococcus* spp., which were typically white/beige.

The two sampling approaches yielded complementary results for bacterial counts. Differences in the numbers of CFUs were analyzed in the NB modeling, and as mentioned above, the sampling time was included in the modeling as the variable “hour”, but this variable was later dropped because it did not reach statistical significance.

The passive sampling allowed for a snapshot of each 1 h over the 4 h of air sampling at the dental clinic, and it provided a better means to observe a higher variety of colony morphotypes since the CFUs were dispersed on the 20 plates. Both methods promoted the growth and isolation of culturable viable mesophilic, aerobic microbes at 37°C, which are a mirror of the typical human oropharynx and URT microbiome of patients undertaking dental treatment in the clinic. The two‐layer protocol for plating out the gelatin filters used in active sampling was developed in this study. The adjustment of the number of passive plates was a result of the NB regression and statistical analysis.

**Figure 4:**
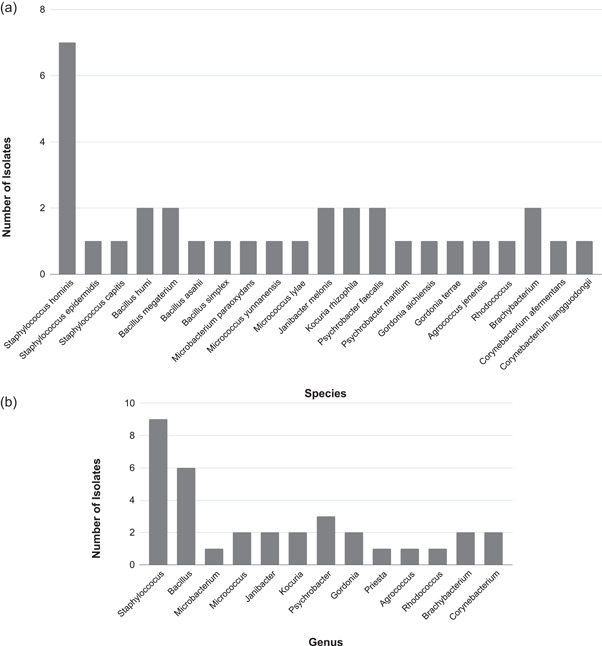
(a) Passive sampling on the first 1 h onto 20 media plates on 24 February 2022; the number of patients = 14; the total number of people in the clinic = 41. There are 21 distinct species from 32 isolates. *Staphylococcus hominis* is the most abundant (*n* = 7). (b) Passive sampling on the first 1 h onto 20 media plates on 24 February 2022; the number of patients = 14; the total number of people in the clinic = 41. There are 13 different genus groups from 32 isolates. *Staphylococcus* spp. are the most abundant (*n* = 9), followed by *Bacillus* spp. (*n* = 6).

Then, the diversity percentage of cultured mesophilic bacteria in passive media plates from the first 1‐h sampling in February 2022 was assessed. Here, the average number of patients undertaking treatment was 14, and the average total number of people in the clinic was 41. Some 35 isolates on 20 passive plates were detected. Of these, two were yeasts based on cell morphology. Of the remaining 33 isolates, some 21 distinct species were identified based on the 16S rRNA gene (Figure 4a). *Staphylococcus hominis* was the most abundant species (22%). There were 13 different genus groups from 32 isolates (Figure 4b). *Staphylococcus species* were the most abundant (*n* = 9), followed by *Bacillus* spp. (*n* = 6). None of the environmental *Bacillus* spp. isolated from this sampling period is known to be a human pathogenic species.

**Figure 5:**
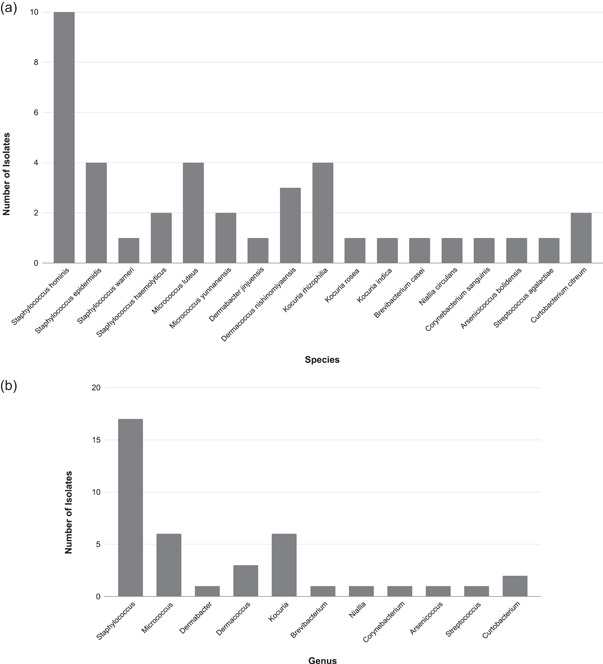
(a) Passive sampling on the fourth 1‐h onto 20 media plates on 17 March 2022; the number of patients = 19; the total number of people in the clinic = 47. There are 17 distinct species from 40 isolates. *Staphylococcus hominis* is the most abundant (*n* = 10). (b) Passive sampling on the fourth 1‐h on 20 media plates on 17 March 2022; the number of patients = 19; the total number of people in the clinic = 47. There are 11 different genera from 40 isolates. *Staphylococcus* spp. are the most abundant (*n* = 17), followed by *Micrococcus* spp. (*n* = 6).

**Figure 6:**
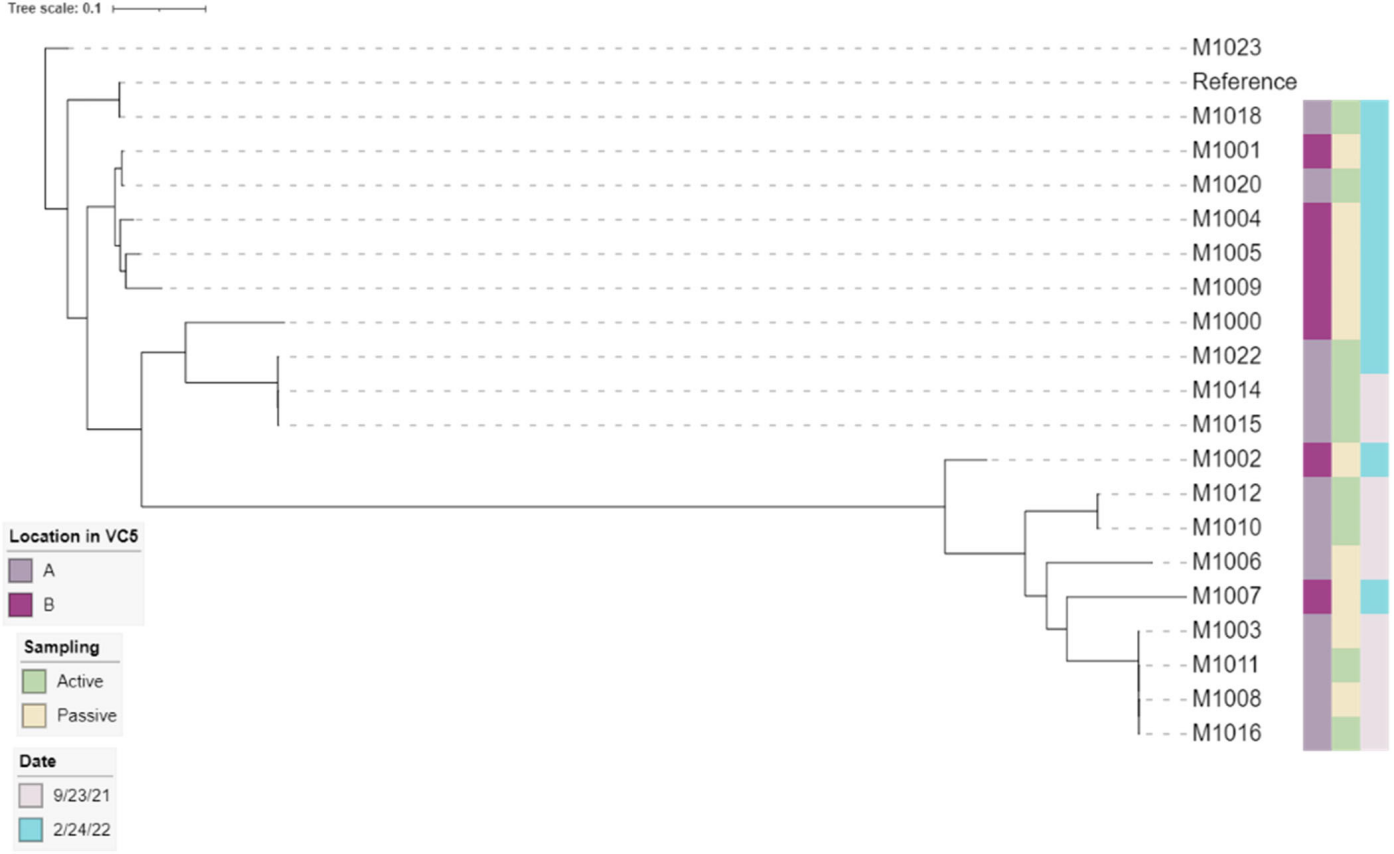
*Staphylococcus hominis* whole genome sequencing (WGS) Dendrogram. It shows four main clusters of *S. hominis* isolated from active and passive samplings from two locations in VC5 on two distinct dates. Those two dates are 5 months apart from each other, for example, 23 September 2021, and 24 February 2022. *S. hominis* M1018 was used to make the reference, and *S. hominis* subsp. *hominis* M1023 is the Type Strain ATCC 27844 that was used to root the RAxML tree generation.

Also, the diversity percentage of cultured mesophilic bacteria in TSA passive media plates from the fourth 1‐h sampling in March 2022 was assessed. During that sampling period, the average number of patients undertaking treatment was 19, and the average total number of people in the clinic was 47. There were 42 isolates in total on the 20 passive plates, 1 was yeast based on cell morphology, and out of the 41 isolates, some 17 distinct species were found from the 16S rRNA gene (Figure 5a). *Staphylococcus hominis* was again the most abundant species (24%). There are 11 different genera from 40 isolates (Figure 5b), and *Staphylococcus* spp. are the most abundant (*n* = 17), followed by *Micrococcus* spp. (*n* = 6). All isolates identified as *S. hominis* underwent whole genome sequencing (Figure 6). This confirmed their initial species identification.

Culture‐based surveillance of the air microbiome may have underestimated species diversity and failed to capture difficult‐to‐grow organisms. While sequence‐based approaches such as 16S amplicon sequencing may provide a more complete assessment of the air microbiome, they may also capture remnant nonviable DNA from organisms. As the focus of this study was to number viable aerobic culturable bacteria to evaluate the effectiveness of far‐UVC irradiation in reducing their number in future studies, therefore, the culture‐based protocols were preferred to culture‐independent 16S amplicon sequencing.

This wide range of bacterial species, which was identified from the samplings, showed the diversity of microbial load in the air microbiome over time and the number of patients in the dental clinic. As suggested earlier, this diversity may not only be due to the number of patients but also to variations in patient diagnoses, dental procedures, as well as individual oral bacterial composition (Sato et al., 2015), which differs significantly between individuals.

A wider variety of 50 species over the months of the study were found using passive sampling plates, while only 14 species were isolated using the active sampling method (Table 3). Most bacterial isolates listed in Table 3, are Gram‐positives, with only two Gram‐negative environmental bacterial species, *Psychrobacter maritimus*, and *Psychrobacter faecalis*. Those two aerobic species were isolated using passive samplings. The passive sampling protocol in this study also collected facultatively anaerobic Gram‐positive bacteria, such as environmental *Arsenicicoccus bolidensis* and native human *Enterococcus faecalis*. Conversely, all species isolated from active sampling were Gram‐positive. It could be argued only Gram‐positive bacteria, which are generally more resistant to desiccation than Gram‐negative bacteria, survived the air‐flow suction through the gelatin membrane in the airports. Also, the lower bacterial diversity on active plates could be due to fungal overgrowth or fungal inhibition of bacteria since cycloheximide was not used on active plating in this study.

**Table 3:** Diversity of bacterial species isolates from active and passive air samplings from September 2021 through March 2022.

Bacterial species identified only in passive samplings	Bacterial species identified in both* active & passive samplings
*Agrococcus jenensis*	Bacillus cereus*
*Arsenicicoccus bolidensis*	Bacillus mycoides*
*Arthrobacter agilis*	Brachybacterium paraconglomeratum*
*Bacillus asahii*	Corynebacterium falsenii*
*Bacillus humi*	Kocuria rhizophila*
*Bacillus megaterium*	Micrococcus endophyticus*
*Bacillus niacin*	Micrococcus luteus*
*Bacillus thuringiensis*	Rhodococcus hoagii*
*Bacillus simplex*	Sporosarcina ureae*
*Brachybacterium huguangmaarense*	Staphylococcus capitis*
*Brevibacterium casei*	Staphylococcus cohnii*
*Corynebacterium afermentans*	Staphylococcus epidermidis*
*Corynebacterium liangguodongii*	Staphylococcus hominis*
*Corynebacterium sanguinis*	Staphylococcus lugdunensis*
*Curtobacterium citreum*	
*Dermabacter jinjuensis*	
*Dermacoccus nishinomiyaensis*	
*Enterococcus faecalis*	
*Gordonia aichiensis*	
*Janibacter melonis*	
*Kocuria indica*	
*Kocuria palustris*	
*Kocuria rosea*	
*Macrococcus equipercicus*	
*Microbacterium paraoxydans*	
*Micrococcus lylae*	
*Micrococcus yunnanensis*	
*Niallia circulans*	
*Psychrobacter faecalis*	
*Psychrobacter maritium*	
*Rhodococcus corynebacterioides*	
*Staphylococcus haemolyticus*	
*Staphylococcus saprophyticus*	
*Staphylococcus schleiferi*	
*Staphylococcus warneri*	
*Streptococcus agalactiae*	

It is worth noting that most species identified in this study are typically found in soil, seawater, sediments, and human skin or gut microbiota, and only a few species have been recently reported in the literature to cause rare infections in people, such as *Corynebacterium sanguinis* (Jaén‐Luchoro et al., 2020), *Dermabacter jinjuensis* (Cho et al., 2018), *Dermacoccus nishinomiyaensis* (Joron et al., 2019; Ogbac, 2021), *Enterococcus faecalis* (Li et al., 2020), *Rhodococcus corynebacterioides* (Kitamura et al., 2012), *Brachybacterium paraconglomeratum* (Murata et al., 2020), *Streptococcus agalactiae* (Ya'qoub et al., 2018), *Micrococcus luteus* (Zhu et al., 2021) and finally, more commonly, *Staphylococcus hominis* (Ahmed et al., 2017; Jeon et al., 2022; Muraki et al., 2022; Uddin et al., 2022; Vasconcellos et al., 2022).

In this study, *S. hominis* comprised a fraction of 60%–80% of all *Staphylococcus* species present in the dental clinic air. In agreement with this study results, Madsen et al. (2018) also found low or no presence of airborne *S. aureus* in living rooms or offices in Greater Copenhagen, with *S. hominis* being the most abundant of the *Staphylococcus* species present in any room. Conversely, *S. aureus* was significantly abundant in samples from nasal cavities or skin from individuals in community households in New York City (Uhlemann et al., 2014). It could be argued that *S. aureus*, typically present in nasal cavities and skin, was in low numbers to be captured by this study air sampling, or that *S. hominis* from the URT was considerably more abundantly in the air due to patients’ respiration. Either way, *S. hominis* seems to be more abundant in the indoor air microbiome of the dental clinic in this study.

To further characterize the most commonly encountered species, *S. hominis*, the whole genome sequencing of cultured isolates from the first and last month of this 6‐month study was performed. Whole genome sequencing and the dendrogram results are shown in Figure 6. MLST typing identified 13 distinct STs. WGS demonstrated that the isolates were overall highly diverse with a few clusters of closely related isolates. These nearly identical isolates were sampled on the same day except for isolates M1022, M1014, and M1015, sampled 6 months apart. Additional longitudinal sampling combined with WGS will provide further insights into the ability of long‐term persistence of specific clones.

### 3.3 *Staphylococcus* spp. susceptibility to 222 nm‐far‐UVC and clonality of *S. hominis*

Since *S. hominis* and *S. epidermidis* were the most abundant species of *Staphylococcus* in this study, their susceptibility to 222 nm‐far‐UVC light was determined in laboratory experiments described above in Section 2.6.

**Figure 7:**
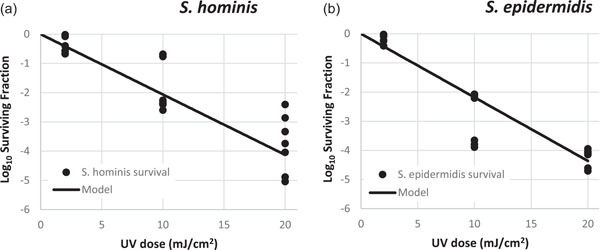
Effect of 222 nm irradiation emitted from a KrCl excimer lamp with a filter on survival of distinct bacterial isolates, using the exponential dose–response model (Equation 1). (a) *Staphylococcus hominis*: rate constant of *k* = 0.475 cm^2^/mJ ± 0.025 (standard error). The susceptibility was evaluated using triplicate samples for each of the three isolates; (b) *Staphylococcus epidermidis*: *k* = 0.502 cm^2^/mJ ± 0.018. The susceptibility was evaluated using triplicate samples for each of the two isolates.

This study results have shown that *S. hominis*, the most abundant *Staphylococcus* sp. in this dental clinic, seems to have a similar susceptibility to 222 nm‐far‐UVC light in laboratory experiments to *S. epidermidis*, also isolated in the dental clinic in this study. When the results of the testing were fit to a log‐linear (exponential) disinfection, the rate constants were determined to be *k* = 0.475 cm^2^/mJ ± 0.025 (standard error) for *S. hominis* and *k* = 0.502 cm^2^/mJ ± 0.018 for *S. epidermidis* (Figure 7). The results suggest that these *Staphylococcus* spp. are similarly susceptible to killing by 222 nm‐far‐UVC. The *k* values for *S. hominis* and *S. epidermidis* in this study are similar to those previously reported in the literature for *S. aureus* using a KrCl lamp, although without the addition of a filter, which measured *k* values in liquid suspension to be between 0.62 and 1.12 cm^2^/mJ (Matafonova et al., 2008). These three *S. hominis* isolates, M1018, M1020, and M1022, have similar susceptibility to 222nm‐far‐UVC, that is, their DNA repair pathways are similar. Conversely, they represent different clones, as shown in Figure 6, and they may also differ in pathogenicity (not evaluated in this study).

## 4 CONCLUSIONS

This study established protocols using two air sampling methods concomitantly to enumerate, isolate and identify baseline aerobic bacteria in the air microbiome of the dental clinic using regression modeling and statistical studies. This study showed persistent isolation of viable and culturable microbes such as *S. hominis*, which are known to be potentially pathogenic. The recommended protocol is for 4 h with active sampling in two locations with samplings times of choice on top of cabinets A and B, and concomitantly for passive sampling 1/1/1 in the same two locations, that is, on top of cabinets A or B.

NB regression outperformed Poisson regression for modeling bacterial abundance data based on AIC. The NB regression model was able to predict bacterial CFU counts relatively consistently across multiple random splits of the data into training and testing halves.

Passive media plates picked up environmental bacteria such as *Bacillus* spp., *Micrococcus* spp., and *Kocuria* spp., among others, but those were in significantly fewer numbers in contrast to the consistently higher numbers of *Staphylococcus* spp. and specifically, *S. hominis*.

A wide diversity of bacterial species isolates was identified, from both active and passive air sampling. The passive sampling protocol provided the means for the isolation of a much higher number and diversity of species, including two facultatively anaerobic bacteria, *Arsenicicoccus bolidensis*, and *Enterococcus faecalis*. This is encouraging as it shows the same protocols can be used to isolate even anaerobic bacteria if isolation plates are incubated under anaerobic conditions. Anaerobic bacteria were not the focus of this study, but they might be of interest in future studies.

*S. hominis* isolates were persistently abundant in both aerobic passive and active sampling protocols. The aerobic air microbiome baseline consisted of *S. hominis* (22%–24%) and comprised 60%–80% of all *Staphylococcus* spp. throughout the months of this study.

These air sampling protocols and confirmation of susceptibility of *S. hominis* to far‐UVC, arising from this study, support the use of 222 nm far‐UVC lamps in dental clinics. Recent studies reported in the literature using controlled experimental settings have suggested that direct use of 222 nm‐far‐UVC irradiation below exposure limits is successful in inactivating pathogens in airborne aerosols (Buonanno et al., 2020; Eadie et al., 2022; Welch et al., 2018, 2022). Conversely, in this study, the focus is a dental clinic, a real environment where variables and conditions are uncontrolled.

One of the future challenges is to incorporate a realistic assessment of the reduction in numbers of viable aerobic bacteria in the air microbiome and their susceptibility to far‐UVC irradiation while patients are receiving dental treatment.

## AUTHOR CONTRIBUTIONS

**Marilena Aquino de Muro**: Conceptualization (equal); Data curation (equal); Formal analysis (equal); Investigation (equal); Methodology (equal); Validation (equal); Writing—original draft (equal); Writing—review & editing (equal). **Igor Shuryak**: Conceptualization (equal); Data curation (equal); Formal analysis (equal); Investigation (equal); Methodology (equal); Validation (equal); Writing—original draft (equal); Writing—review & editing (equal). **Anne‐Catrin Uhlemann**: Conceptualization (equal); Data curation (equal); Formal analysis (equal); Investigation (equal); Methodology (equal); Validation (equal); Writing—original draft (equal); Writing—review & editing (equal). **Alice Tillman**: Data curation (equal); Formal analysis (equal); Methodology (equal); Writing—review & editing (equal). **Dwayne Seeram**: Data curation (equal); Formal analysis (equal); Methodology (equal); Writing— review & editing (equal). **Joseph Zakaria**: Methodology (supporting). **David Welch**: Writing—review & editing (equal). **Steven M Erde**: Project administration (supporting). **David J Brenner**: Conceptualization (equal); Project administration (lead); Resources (lead); Supervision (lead).

## CONFLICT OF INTEREST STATEMENT

The authors declare no conflict of interest.

## ETHICS STATEMENT

Protocols and procedures employed in this investigation were reviewed and approved by the appropriate institutional review committees.

## Data Availability

All data are provided in full in the results section of this paper apart from the WGS DNA sequences encompassing this study *Staphylococcus hominis* isolates which are available at https://www.ncbi.nlm.nih.gov/bioproject/PRJNA895435.

## Appendix

**Supplementary Methods**

**Regression Modelling and Statistical Analysis**

The data collected from air sampling were compiled and imported into R 4.2.0 software for regression analysis (R Core Team, 2017). The dependent variable (outcome) was bacteria_CFUs (colony counts per sample). The sampling time was included in the modeling as the variable "hour", but this variable was later dropped because it did not reach statistical significance.

The independent (predictor) variables were sampling (active or passive), month (calendar month of samplings), weekday (Tuesday or Thursday), hour (hour of samplings, 1, 2, or 3), location (on top of cabinet A or B) and people (average number of people in the room). The goal of the analysis was to construct a model for predicting the outcome variable (bacterial abundance) using the predictor variables.

The data set was randomly split into training and testing halves. Model fitting and selection was performed on the training half, and evaluation of performance using a coefficient of determination (R2), root mean squared error (RMSE), and mean absolute error (MAE) metrics was done on the testing half. Since the outcome data are integer counts, Poisson and Negative Binomial (NB) regressions were considered as the analysis methods. Poisson regression was performed using the glm R function, and NB regression was performed using the glm.nb function in the MASS R package. NB regression outperformed Poisson regression based on Akaike information criterion (AIC) values (Burnham & Anderson, 2002), so it was used for more detailed analyses.

The NB regression utilized here is a generalized linear model (GLM), where the predicted mean outcome was assumed to depend linearly on each predictor variable. However, a log link function was used, so the model is fitted on a natural log scale, i.e., the predictions were ln[mean] and the model parameters (fitted coefficients) were on the ln scale as well. The effect of each predictor was therefore additive on the log scale, but multiplicative on the linear scale.

Model selection was performed on NB regression variants to identify the best‐supported model. The initial NB regression attempt on the training data included all relevant predictors: sampling, location, month, weekday, hour, and people. To obtain the best‐supported and simplified model, non‐significant predictor variables were sequentially removed and resulting model variants were compared using AIC values and likelihood ratio tests (Burnham & Anderson, 2002). We tested for potential multicollinearity between predictor variables using variance inflation factors (VIF) using the VIF R function in package regclass. Potential interactions between the remaining variables were also evaluated.
